# Stronger Drought Response of CO_2_ Fluxes in Tundra Heath Compared to *Sphagnum* Peatland in the Sub‐Arctic

**DOI:** 10.1111/gcb.70210

**Published:** 2025-04-24

**Authors:** Valentin Heinzelmann, Julia Marinissen, Rien Aerts, J. Hans C. Cornelissen, Stef Bokhorst

**Affiliations:** ^1^ Amsterdam Institute for Life and Environment, Section Systems Ecology Vrije Universiteit Amsterdam Amsterdam the Netherlands

**Keywords:** climate change, CO_2_ fluxes, drought, extreme events, peatland, plant responses, sub‐arctic ecosystems

## Abstract

Drought events are increasing in frequency and intensity due to climate change, causing lasting impacts on plant communities and ecosystem functioning. In the sub‐arctic, climate is changing at a rate above the global average with amplifying effects on the carbon cycle. Drought‐induced shifts in the balance between productivity and respiration might have important implications for climate change feedbacks in these regions. However, little is known about how carbon fluxes in sub‐arctic ecosystems respond to drought, hampering predictions. Here, we test how two important but contrasting sub‐arctic ecosystem types, *Sphagnum* peatland and tundra heath, respond to experimental drought. Mesocosms were exposed to a full precipitation exclusion for 7 weeks, decreasing gravimetric water content by 66% and 53% for *Sphagnum* peatland and tundra heath, respectively. Drought suppressed all CO_2_ flux components. Gross primary productivity was on average reduced by 47% and 64%, and ecosystem respiration by 40% and 53% in *Sphagnum* peatland and tundra heath, respectively. Concomitantly with the ecosystem fluxes, leaf photosynthesis of the three most abundant vascular plant species per ecosystem type was on average suppressed by 40% (peatland) and 77% (tundra heath). Drought resulted in high plant mortality, with up to 54% (peatland) and 73% (tundra heath) dead shoots, which might represent a significant legacy effect suppressing CO_2_ uptake in subsequent growing seasons. In summary, tundra heath was overall more responsive to drought than peatland. This differential sensitivity, previously unaccounted for, might be important in the future under intensifying drought events. Considering that tundra heath covers more than half of the sub‐arctic land area, its drought responsiveness might induce significant reductions in total arctic net CO_2_ uptake. This would move the arctic carbon balance further toward a net CO_2_ source.

## Graphical abstract

Drought events cause lasting impacts on plant communities and carbon cycling in the sub‐arctic. Under experimental drought, tundra heath showed stronger reductions in CO_2_ uptake and higher plant mortality than Sphagnum peatland. This differential sensitivity, previously unaccounted for, might be important under intensifying drought events and could significantly reduce total arctic net CO_2_ uptake.

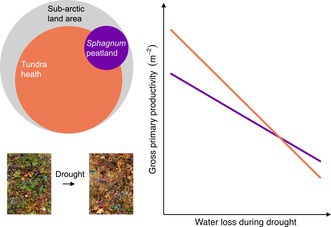

## 1 Introduction

Climate is changing at its fastest rate at northern latitudes, where surface temperatures have risen about two to four times above the global average (AMAP 2021; Meredith et al. 2022; Rantanen et al. 2022). Alongside gradual warming, extreme weather events are occurring more frequently in arctic regions (Walsh et al. 2020) and are manifested during summer as droughts or heatwaves (Rantanen et al. 2024; Rietze et al. 2024). Given the huge amount of soil organic carbon stored in the arctic land surface (Schuur et al. 2022; Tarnocai et al. 2009), any changes in climate or weather conditions might affect feedbacks of relevance to the global carbon cycle (Dorrepaal et al. 2009; Maes et al. 2024; Schuur et al. 2022). Owing to the close coupling between the carbon and water cycle (Gentine et al. 2019), drought is expected to have major impacts on ecosystem functioning. Drought is meteorologically defined as a time of below‐average precipitation (Slette et al. 2019) and is forecasted to become more frequent and intense (IPCC 2023). If soil moisture (i.e., gravimetric soil water content) under drought falls below a critical threshold, ecosystems become water‐limited (Wankmüller et al. 2024) which dampens plant and microbial metabolism and consequently suppresses ecosystem‐level CO_2_ fluxes (Ciais et al. 2005; van der Molen et al. 2011). CO_2_ uptake by plants, expressed as gross primary productivity (GPP) at the ecosystem level, is typically more strongly affected by drought than CO_2_ losses through ecosystem respiration (Reco, the sum of autotrophic and heterotrophic respiration). This imbalance in flux reductions leads to a decrease in net ecosystem exchange (NEE, the relatively small difference between GPP and Reco; Schwalm et al. 2010; Shi et al. 2014), resulting in a feedback between drought and climate change (Seneviratne et al. 2010). Apart from these overall patterns, drought impacts are ecosystem‐specific, thus leading to differential sensitivities (Huxman et al. 2004; Knapp et al. 2024; Teuling et al. 2010). Drought sensitivity can be defined as response in CO_2_ fluxes under proceeding drought. It is controlled by (a) atmospheric conditions, (b) plant traits, and to a large degree by (c) soil properties (i.e., water content and soil texture; Fu et al. 2022; Wankmüller et al. 2024). Initial water content represents a reservoir for plant water demand and thus defines a margin of drought tolerance until ecosystems become water‐limited. In the sub‐arctic, low water contents are found over a large area in the ecosystem‐type tundra heath (5.2 Mkm^2^), whereas water‐rich areas like *Sphagnum*‐dominated peatlands are smaller (0.86 Mkm^2^, Ramage et al. 2024). The contrasting water content characteristics between the two ecosystem types likely determine their drought responses; empirical evidence is, however, lacking.

Besides soil water content, plant traits shape ecosystem‐level responses to drought (Anderegg et al. 2018; Helbig et al. 2020; Teuling et al. 2010). Vascular plants, which form the dominant species group in tundra heath, have clear drought‐coping mechanisms. In general, stomatal aperture is adjusted to reduce water loss at the cost of reduced leaf photosynthesis (Fu et al. 2022) and, consequently, ecosystem GPP. If drought continues and the wilting point is reached, plants might die from carbon starvation or hydraulic failure (McDowell et al. 2008). *Sphagnum* mosses, on the contrary, which co‐exist with vascular plants (Le et al. 2022) in peatlands, lack clear mechanisms to reduce water loss. As in vascular plants, the photosynthesis of mosses is reduced under drought (Strack and Price 2009), but primarily controlled by water content and not by morphological adjustments (i.e., regulation in stomatal aperture). To avoid drought stress, *Sphagnum* mosses have evolved traits that allow them to store and retain great amounts of water. Hyaline cells, for example, are effective water storage units (Hayward and Clymo 1982), while the branches and leaves of mosses effect a matrix of high water holding capacity (Bengtsson et al. 2020), creating a moist environment. In short, mosses avoid drying out by retaining a lot of water. The interplay between water content and dominant plant communities with their respective traits will ultimately define ecosystem drought sensitivities. To understand this interplay, the dwarf shrub *Empetrum hermaphroditum* plays a special role, as it is common to both ecosystem types and can therefore be used to assess the plant drought response at broadly similar trait make‐up.

*Sphagnum*‐dominated peatland and tundra heath contribute to arctic growing season NEE with 54 and 358 Tg CO_2_‐C respectively (Ramage et al. 2024). This amount is equivalent to 21%–82% of the expected annual CO_2_‐C emissions from the arctic by 2100 (Schuur et al. 2022) which illustrates the importance of the two ecosystem types in the arctic carbon balance. Therefore, the aim of the present paper is to quantify how CO_2_ fluxes in *Sphagnum* peatland and tundra heath respond to drought. To do so, mesocosms from both ecosystem types were exposed to a 7‐week‐long precipitation exclusion. We hypothesize (i) that drought will suppress CO_2_ fluxes (both GPP and Reco) more strongly in tundra heath than in *Sphagnum* peatland. (ii) For the underlying mechanisms, we expect that all vascular plant species will reduce leaf‐level CO_2_ fluxes and that the response of the overlapping plant species *E. hermaphroditum* will be more pronounced in tundra heath compared to peatland. (iii) Additionally, we expect that vascular plant mortality will be higher in tundra heath than in peatland.

## 2 Material and Methods

### 2.1 Mesocosms and Experimental Design

The experimental drought study was conducted in an experimental garden of the Abisko Scientific Research Station (68**°**36′ N, 18**°**83′ E), Northern Sweden during the summer of 2022. Mean annual temperature at the station is 0.5°C (1990–2021) and mean annual precipitation is 349 mm (1990–2021). Mean summer (averaged over June, July, and August) temperature during the experiment was 11°C and precipitation was 147 mm (SMHI 2025).

A total of 32 mesocosms (2 ecosystem types × 2 treatments × 8 replicates) were sampled from representative peatland and tundra heath ecosystems at the end of June 2022. Sampling was conducted in a block design with experimental drought and control mesocosms collected from each block (8 blocks × 2 paired samples per ecosystem type) to ensure equivalent environmental conditions and vegetation composition. To collect vegetation and soil, including the major roots as a complete layer, the mesocosms (39 × 28 cm) were cut to approximately 15 cm depth, taken out by hand, and placed in plastic trays. Peatland mesocosms were obtained from a *Sphagnum fuscum* (Schimp., H.Klinggr.) dominated site in Stordalen (68°35′ N, 19°04′ E). The heath mesocosms were obtained from tundra heath, near Abisko Scientific Research Station.

After sampling in the field, experimental drought was induced by placing the mesocosms underneath sloping (approximately 45°, facing west) plexiglass panels which ensured full removal of all summer precipitation. With the present study, we aimed to understand mechanisms of drought sensitivity in two ecosystem types rather than reconstructing past drought events, following (De Boeck et al. 2020). Measuring responses over a range of soil water contents (inversely related to drought intensities) might provide important process information for Earth system models, which can then be adjusted to different climate scenarios. Furthermore, future climate extremes are predicted to become more intense and are likely to exceed past records (Fischer et al. 2021; IPCC 2023), which supports testing for extreme drought. We therefore opted for a drought regime that extended beyond historical drought episodes (Figure S1). The length of 7 weeks was ultimately determined during the experiment and based on the observation of severe drought impacts (such as high plant mortality).

Drought was initiated on 24.06.22 (tundra heath) and on 27.06.22 (*Sphagnum* peatland) and lasted until 12.08.22. The experimental drought group was kept in trays with nine holes (6 mm) in the bottom to allow for seepage water loss leaving the trays. Control treatments were regularly watered (see below) and their trays were intact.

#### 2.1.1 Mesocosm Water Content

Each mesocosm was weighed before placement under the rainout shelters and reweighed every 3–4 days for the duration of the experiment. Total weight decline over time was attributed to water loss (evapotranspiration or seepage). The control group was kept at its initial weight by re‐watering every 3–4 days with tap water (which has very low nutrient concentrations in Abisko). The gravimetric water content of the mesocosms was estimated by sampling additional soil cores (diameter = 10 cm, height between 11 and 14 cm, *n* = 8 per ecosystem type) from each block and subsequent oven‐drying (72 h at 70°C). We assumed that the measured water content of each soil core is representative of each experimental block. Weight loss of the soil mesocosms was then used to calculate their gravimetric water content over time. Mesocosms were weighed 11 (peatland) and 12 (tundra heath) times during the experiment.

### 2.2 CO_2_ Flux Measurements and Calculations

To quantify the effects of drought on ecosystem carbon fluxes, we measured the net CO_2_ flux (Net Ecosystem Exchange, NEE) and release (Ecosystem Respiration, Reco).

Measurements were conducted by placing a clear Perspex chamber (diameter = 21 cm, height = 10 cm, volume = 3464 cm^3^; the chamber thus covered approximately 1/3 of the total surface area of the mesocosm) over the vegetation and monitoring the rate of change in CO_2_ concentrations, using an infrared gas analyzer (IRGA; EGM‐5 PP‐systems). To minimize internal chamber air exchange with the outside, a rubber band was attached at the open bottom perimeter of the chamber, together with a plastic skirt weighed down with chains. A small fan was attached inside the chamber to mix the air (Street et al. 2007).

Flux measurements were conducted on a weekly basis with an interruption of 2 weeks in mid‐July, resulting in five measurements for each ecosystem type. Mesocosms were removed from the rain shelters and placed on a nearby table. Measurements started with a transparent chamber to measure NEE. Subsequently, the chamber was lifted off the mesocosm to ventilate the chamber. The chamber was then put back, covered with an opaque cloth blocking out all sunlight, and Reco was measured. The accumulation time for each component was 2 min; CO_2_ concentrations were recorded every second. For each measurement (NEE and Reco), the concentration was linearly regressed on time, and the slope of the fitted model (concentration change over time) was used in the following equation:

(1) $$F_{\mathrm{CO}2}=\left(\frac{∆{\mathrm{CO}}_{2}}{∆t}\times T_{i}\times P\right)\times {\left(V_{i}\times T\times P_{i}\right)}^{-1}\times \frac{V}{A}\times 1000$$

where $F_{\mathrm{CO}2}$ = flux of CO_2_ (μmol × m^−2^ × s^−1^), $\frac{∆{\mathrm{CO}}_{2}}{∆t}$ = temporal CO_2_ concentration change (ppm × s^−1^), Δ*t* = change in time (s^−1^), slope of linear regression either for NEE or Reco, *T*_*i*_ = standard temperature (273.15 K), *P* = pressure (kPa), *V*_*i*_ = standard volume (22.4 dm^3^/mol), *T* = ambient air temperature (K), *P*_*i*_ = standard pressure (101.325 kPa), *V* = volume of the chamber (m^3^) and *A* = area of the flux chamber (m^2^).

Pressure *P* was derived for each measurement interval by averaging pressure values logged at 1/60 Hz by the IRGA. Air temperature was measured by a temperature probe (HOBO S‐TMB‐M0xx 12‐Bit), which was covered by a radiation shield and connected to a data logger (H21‐002 HOBO Micro Station). The probe was mounted 5 cm above the mesocosm and logged at 1 min intervals during its corresponding CO_2_‐flux measurement. Furthermore, we measured soil temperature (at 5 cm depth) using a handheld thermometer (Hanna Instruments 935005k‐thermocouple with the thermocouple probe HI766E1).

Gross primary productivity was calculated as:

(2) GPP=Reco−NEE

Fluxes from the atmosphere to the ecosystem (C gains) were defined as positive, NEE and Reco were therefore multiplied by −1 after using Equations (1) and (2). Since weighing of the mesocosms was done at a different time than the flux measurements, soil water content on those days was estimated based on the nearest weighing date or the mean between two nearest days.

To estimate plant species composition during the later part of the growing season (first week of August), we used the point intercept method (Jonasson 1988). Each mesocosm was placed under a metal frame holding a movable bar with seven horizontally aligned holes, each 2.5 cm apart. The bar was moved in four intervals over the mesocosm, leading to a total of 28 points per mesocosm. A metal pin of 0.5 cm diameter was lowered through these holes at each step until it hit the ground. Each plant species that touched the pin was recorded (Table S1).

#### 2.2.1 Leaf‐Level CO_2_ Flux and Water Content

Plant leaf‐level CO_2_‐fluxes were measured during the 5th week of drought (end of July), using one leaf or shoot from each of the three most dominant vascular plant species in each mesocosm. Sampled species included *Rubus chamaemorus* L., *Empetrum hermaphroditum* L., and *Andromeda polifolia* L. in *Sphagnum* peatland, and *E. hermaphroditum, Vaccinium uliginosum* L., and *Arctostaphylos alpina* L. in tundra heath. Branches were sampled for *E. hermaphroditum* and *A. polifolia* due to their small leaf size. Leaves and branches were randomly selected from an area of the mesocosm lying outside the location attributed to the CO_2_ flux measurements. Leaf CO_2_ fluxes were measured immediately after sampling and the procedure followed the method described for whole mesocosm fluxes except that a small transparent tube (8 cm length, 2.2 cm diameter, *V* = 30.4 cm^3^) was used. CO_2_ fluxes were calculated using Equation (1); however, the division by area was replaced with the corresponding leaf dry weight (see below). Leaf photosynthesis was calculated according to Equation (2).

Fresh weight of the leaves was quantified within 30 min of leaf CO_2_ flux measurements. For *E. hermaphroditum* and *A. polifolia* , leaves were separated from the stem. The leaves were then oven‐dried (70°C, 72 h) and their dry weight was determined. Leaf water content (LWC) was calculated as follows:

(3) $$\mathrm{LWC}=\left(\frac{\text{fresh weight}-\mathrm{dry}\;\text{weight}}{\mathrm{dry}\;\text{weight}}\right)*100$$

Leaf water content of *E. hermaphroditum* was determined for a second time at the end of the experiment (after 7 weeks of drought).

#### 2.2.2 Plant Mortality

To quantify plant mortality, individual plant shoots were tagged at the start of the experiment (*n* = 10 per species) and checked on their alive/dead status at the end of the experiment. Shoots were classified as dead if all leaves had fully turned brown or had fallen off. In *Sphagnum* peatland, we tagged shoots of *E. hermaphroditum, R. chamaemorus*, and *A. polifolia* , and in tundra heath, *E. hermaphroditum, V. uliginosum*, and *A. alpina* .

### 2.3 Drought Sensitivity and Statistical Analyses

To compare drought sensitivity between the two ecosystem types, that is, *Sphagnum* peatland and tundra heath, logarithmic response ratios were calculated. The approach was adapted from Hedges et al. (1999) and calculated as follows:

(4) $$\mathrm{Ln}R_{\text{parameter},\mathrm{it}}=\mathrm{Ln}\left(\frac{{\text{parameter}}_{\text{drought},\mathrm{it}}}{{\text{parameter}}_{\text{control},\mathrm{it}}}\right)$$

with Ln being the natural logarithm of the *i*th pair of blocks (one of eight mesocosm pairs per ecosystem type) at time point *t* (one of five flux measurement occasions); parameter included: mesocosm fluxes (GPP, Reco), gravimetric water content and leaf‐level fluxes ( *E. hermaphroditum* only). To avoid 0‐values for the log‐response ratio calculations, we added 0.01 to mesocosm‐level fluxes and water content and 0.001 to leaf‐level fluxes. Drought sensitivity was thus defined as the logarithmic proportion of CO_2_ flux under drought to CO_2_ flux under control conditions. Small values (i.e., more negative) indicate a stronger drought response.

Relative water loss between the two ecosystem types was compared by using the following equation:

(5) $$\text{Water}\;{\text{loss}}_{\mathrm{it}}=\frac{{\text{water content}}_{\text{control},\mathrm{it}}-{\text{water content}}_{\text{drought},\mathrm{it}}}{{\text{water content}}_{\text{control},\mathrm{it}}}*100$$

Water loss was calculated for each *i*th pair of blocks at time point *t* (one of the moments where weight was measured and water added).

In addition to the Ln response ratio (Equation 4), the following equation was used to express drought effects on CO_2_ fluxes:

(6) $$\text{Relative difference}=\frac{{\text{mean flux}}_{\text{control}}-\text{mean}\;{\text{flux}}_{\text{drought}}}{{\text{mean flux}}_{\text{control}}}*100$$

All statistical analyses and plotting of data were performed in the *R* statistical environment (R Core Team 2023) and Rstudio (Posit Team 2023). The ggplot2 package (Wickham 2016) was used to plot the data. Two‐way repeated‐measures ANOVA was used to test the effects of ecosystem type and time on the relative water loss from the mesocosms. Linear mixed effect models (LMM's) were used to test the effects of the drought treatment and time on water content of mesocosms, on GPP and Reco for each ecosystem type separately with block as random effect. To compare the drought sensitivity of the flux components (GPP and Reco) between the two ecosystem types, separate LMMs were fitted for the Ln response of GPP and Reco. Ecosystem type (peatland or tundra heath), flux component (GPP or Reco) and the Ln response of water content (inversely indicative of drought intensity) were used as fixed effects, block as random effect. Additionally, to test if the drought sensitivity of the two flux components GPP and Reco differed, another LMM was fitted where the Ln response ratios of GPP and Reco are considered as one variable, alongside the same fixed effects as in the first models (i.e., ecosystem type and Ln response of water content). LMM was furthermore fitted on *E. hermaphroditum* Ln response ratios with flux component and ecosystem type as fixed effects and block as random effect. LMM's with species and treatment as fixed effects were fitted on leaf CO_2_ flux components and water content. This was done per ecosystem type and separately for the components of photosynthesis and respiration. LMMs with species, time, and treatment as fixed effects were performed separately on the mean alive shoots of selected plant species in each of the two ecosystem types. The models were conducted with the “lme” command from the “nlme” package (Bates and Bates 2023). *p*‐values were obtained with the “anova” command in R; fixed effects were considered significant at *p* < 0.05. Post hoc comparisons of treatment means were performed using the Tukey method in the “emmeans” package (Lenth 2023).

## 3 Results

**FIGURE 1:**
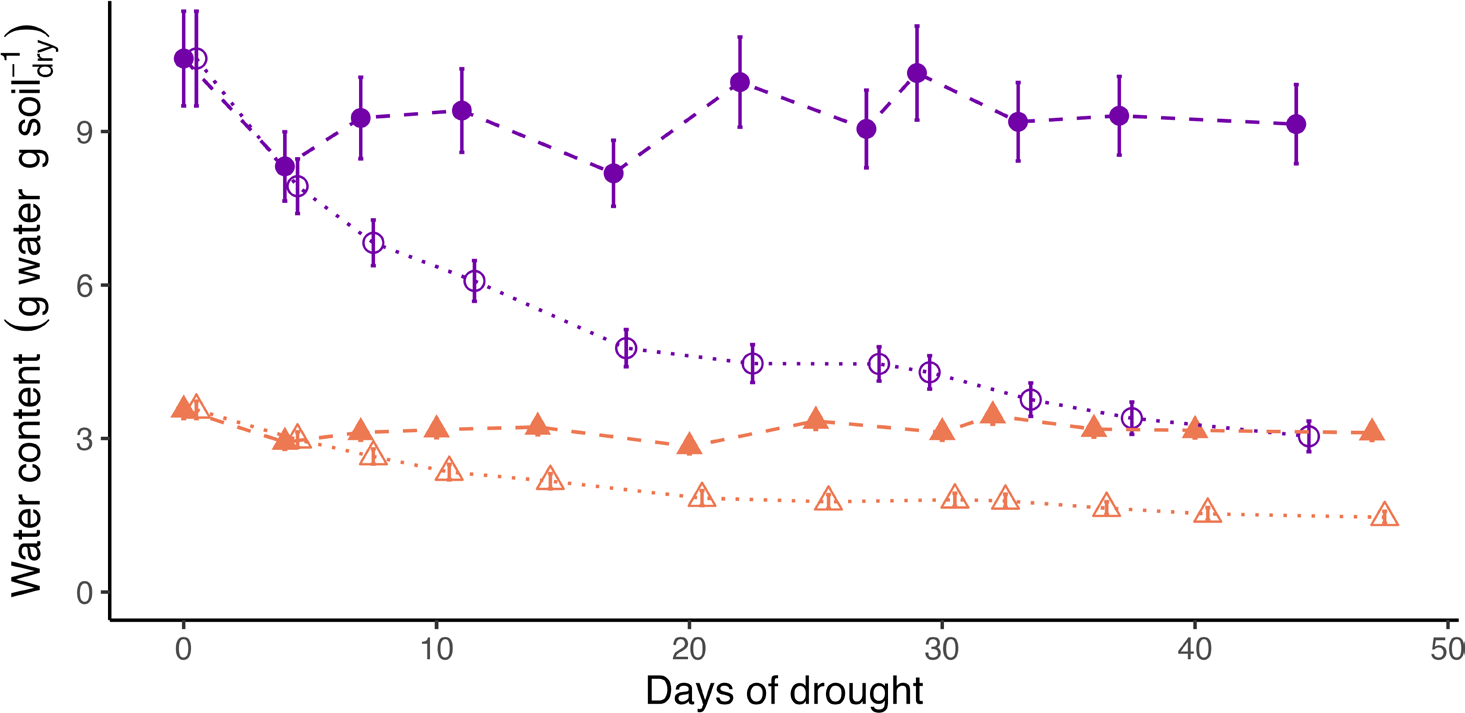
Water content (mean ± standard error, *n* = 8 replicate mesocosms) of sub‐arctic Sphagnum peatland (purple circles) and tundra heath (orange triangles) mesocosms under experimental drought (dotted lines) and control (dashed lines) conditions.

*Sphagnum* peatland had a threefold higher initial water content (Figure 1), and higher absolute and relative water losses compared to tundra heath (Table 1). Drought‐affected water content after 1 week in both ecosystems (Table 2). At the end of the experiment, peatland had lost 66% of its water content and exceeded the water loss of 53% in tundra heath (Figure 1).

**TABLE 1:** Two‐way ANOVA results testing for effects of ecosystem type and time on relative water loss under experimental drought.

	Ecosystem type		Time		Ecosystem type × Time	
	*F*	*p*	*F*	*p*	*F*	*p*
Relative water loss	44.8	< 0.001	67.4	< 0.001	1.7	0.180

*Note:* There were 16 replicate mesocosms for Sphagnum peatland and tundra heath, each with *n* = 8 allocated to drought and *n* = 8 to control watering. Water content was quantified 11 (peatland) to 12 (tundra heath) times during the experiment. numDF, denDF: Ecosystem type = 1, 161; Time = 19, 161; Ecosystem type × Time = 2, 161.

**TABLE 2:** Summary of linear mixed effect model results testing the effect of time and treatment on *Sphagnum* peatland and tundra heath water content and flux components.

Response variable	Ecosystem type	Time		Drought		Time × Drought	
		*F*	*p*	*F*	*p*	*F*	*p*
Water content	Peatland	23.47	< 0.001	647.61	< 0.001	18.65	< 0.001
	Tundra heath	125.4	< 0.001	3277.9	< 0.001	96.9	< 0.001
GPP	Peatland	6.1	< 0.001	99.6	< 0.001	14.2	< 0.001
	Tundra heath	11.7	< 0.001	209.2	< 0.001	9.5	< 0.001
Reco	Peatland	56.4	< 0.001	87.5	< 0.001	13.7	< 0.001
	Tundra heath	53.6	< 0.001	180.5	< 0.001	13.4	< 0.001
NEE	Peatland	18.1	< 0.001	34.8	< 0.001	8.8	< 0.001
	Tundra heath	38.3	< 0.001	69	< 0.001	2.8	< 0.05

*Note:* There were 16 replicate mesocosms per ecosystem type, each with *n* = 8 allocated to control watering or to drought. Water content was quantified 11 (peatland) to 12 (tundra heath) times during the experiment. Fluxes were measured five times during the experiment. numDF, denDF: water content peatland = 10, 147; water content tundra heath = 11, 161; fluxes peatland = 4, 61; and fluxes tundra heath = 4, 63.

**FIGURE 2:**
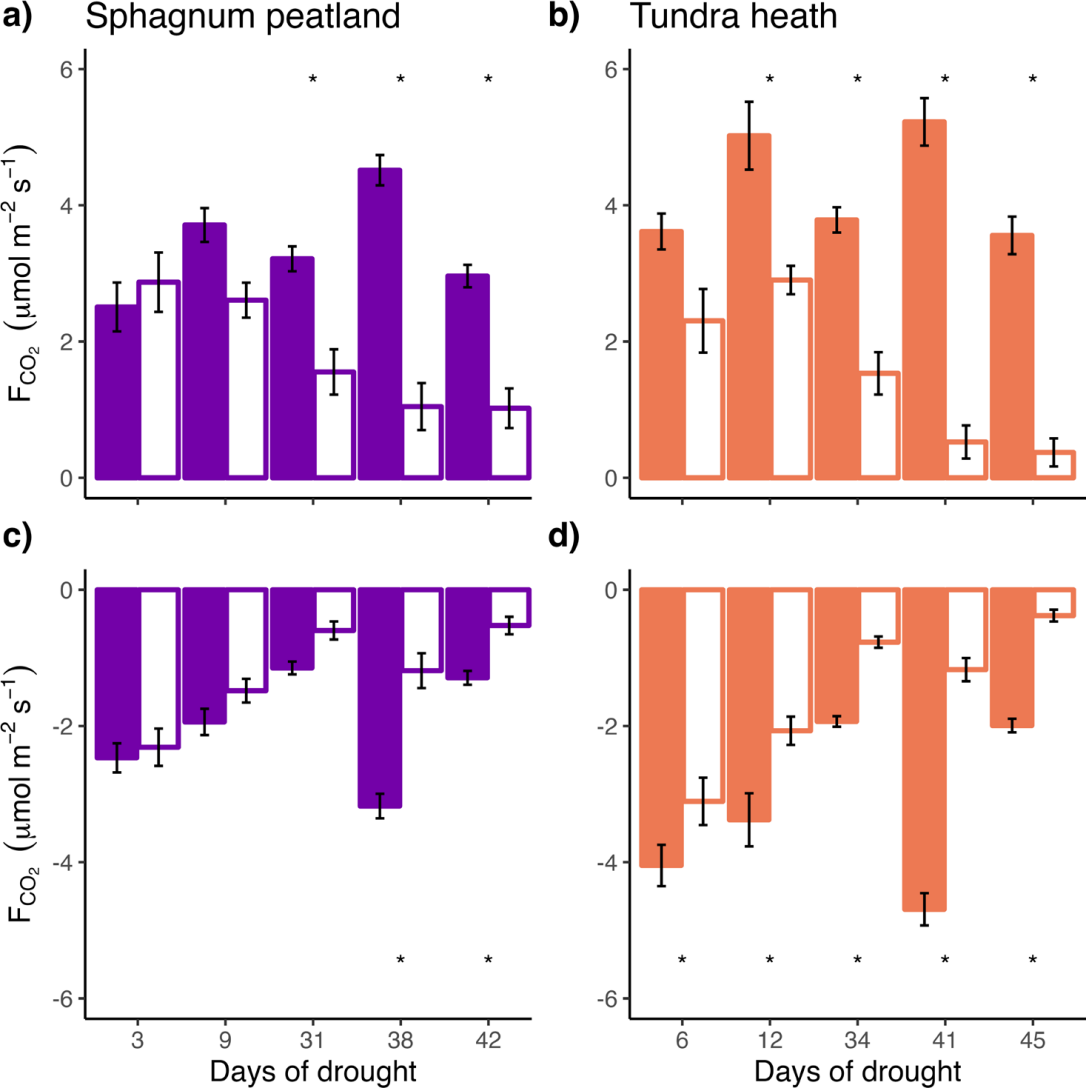
Mean CO_2_ fluxes (± standard error, *n* = 8) of Sphagnum peatland (purple) and tundra heath (orange) mesocosms under control (colored bars) and experimental drought (white bars) conditions. (a, b) Show GPP, positive values mean CO_2_ uptake by the mesocosm. (c, d) Indicate Reco, negative values show CO_2_ release. Asterisks indicate significant differences between control and drought groups (Tukey, *p* < 0.05).

**FIGURE 3:**
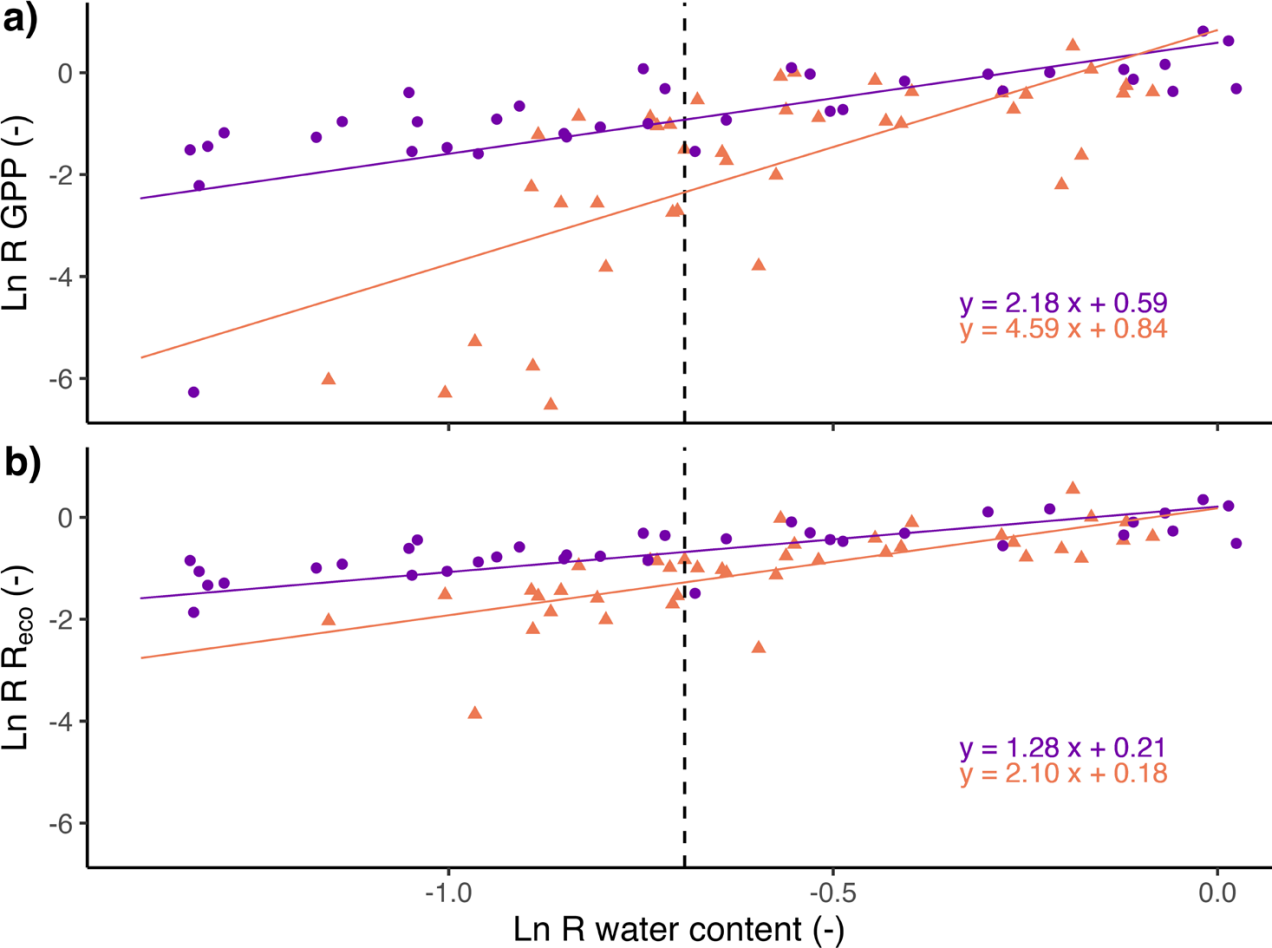
CO_2_ flux response to experimental drought in sub‐arctic Sphagnum peatland (purple circles) and tundra heath (orange triangles). (a) Ln response ratio of GPP and (b) Ln response ratio of Reco correlated with Ln response ratio of water content (*x*‐axis). Each symbol represents the Ln response ratio for a pair of mesocosms from control and drought group (eight paired mesocosm across five measurements per ecosystem type). Trend lines indicate correlations between fluxes with water content (*p* < 0.05). The dashed vertical line corresponds to a 50% decline in water content.

In both ecosystem types, all CO_2_ flux components (GPP, Reco) were suppressed by drought with increasing impacts over time (Figure 2, Table 2). The increase in Reco at the end of the measurement period was associated with an 8°C–10°C increase in soil temperature (Figure 2, Figure S2). Tundra heath CO_2_ fluxes were more sensitive to drought than peatland fluxes, as indicated by the Ln response ratios (Figure 3, Table 3). In both ecosystem types, GPP was more sensitive to drought than Reco, particularly with increasing drought severity over time (Table S2). Relative changes based on mean fluxes of control and drought groups were of significant magnitude and most evident for mesocosm NEE (Table 4).

**TABLE 3:** Summary of linear mixed effect model results testing the effect of ecosystem type (*Sphagnum* peatland or tundra heath) and Ln response ratio of water content on the Ln response ratio of mesocosm CO_2_ fluxes (GPP or Reco).

Response variable	Ecosystem type		Ln response water content		Ecosystem type × Ln response water content	
	*F*	*p*	*F*	*p*	*F*	*p*
Ln R GPP	8.8	0.004	64.6	< 0.001	9.4	0.003
Ln R Reco	5.3	0.025	81.5	< 0.001	4.2	0.045

*Note:* There were 16 replicate mesocosms per ecosystem type, each with *n* = 8 allocated to control watering or drought. Fluxes were measured 5 times during the experiment. numDF, denDF: Ln R GPP and Ln R Reco = 1, 68.

**TABLE 4:** Mean CO_2_ flux components (± standard error) under control and experimental drought conditions.

Flux component	Ecosystem type	Control flux (μmol m^−2^ s^−1^)	Drought flux (μmol m^−2^ s^−1^)	Difference (%)
GPP	*Sphagnum* peatland	3.4 (±0.2)	1.8 (±0.2)	47
GPP	Tundra heath	4.2 (±0.2)	1.5 (±0.2)	64
Reco	*Sphagnum* peatland	−2 (±0.1)	−1.2 (±0.1)	40
Reco	Tundra heath	−3.2 (±0.2)	−1.5 (±0.2)	53
NEE	*Sphagnum* peatland	1.3 (±0.2)	0.6 (±0.1)	57
NEE	Tundra heath	1 (±0.2)	0 (±0.1)	98

*Note:* Drought lasted 45 days on sub‐arctic *Sphagnum* peatland and 48 days on tundra heath.

### 3.1 Leaf CO_2_ Fluxes and Water Content

**FIGURE 4:**
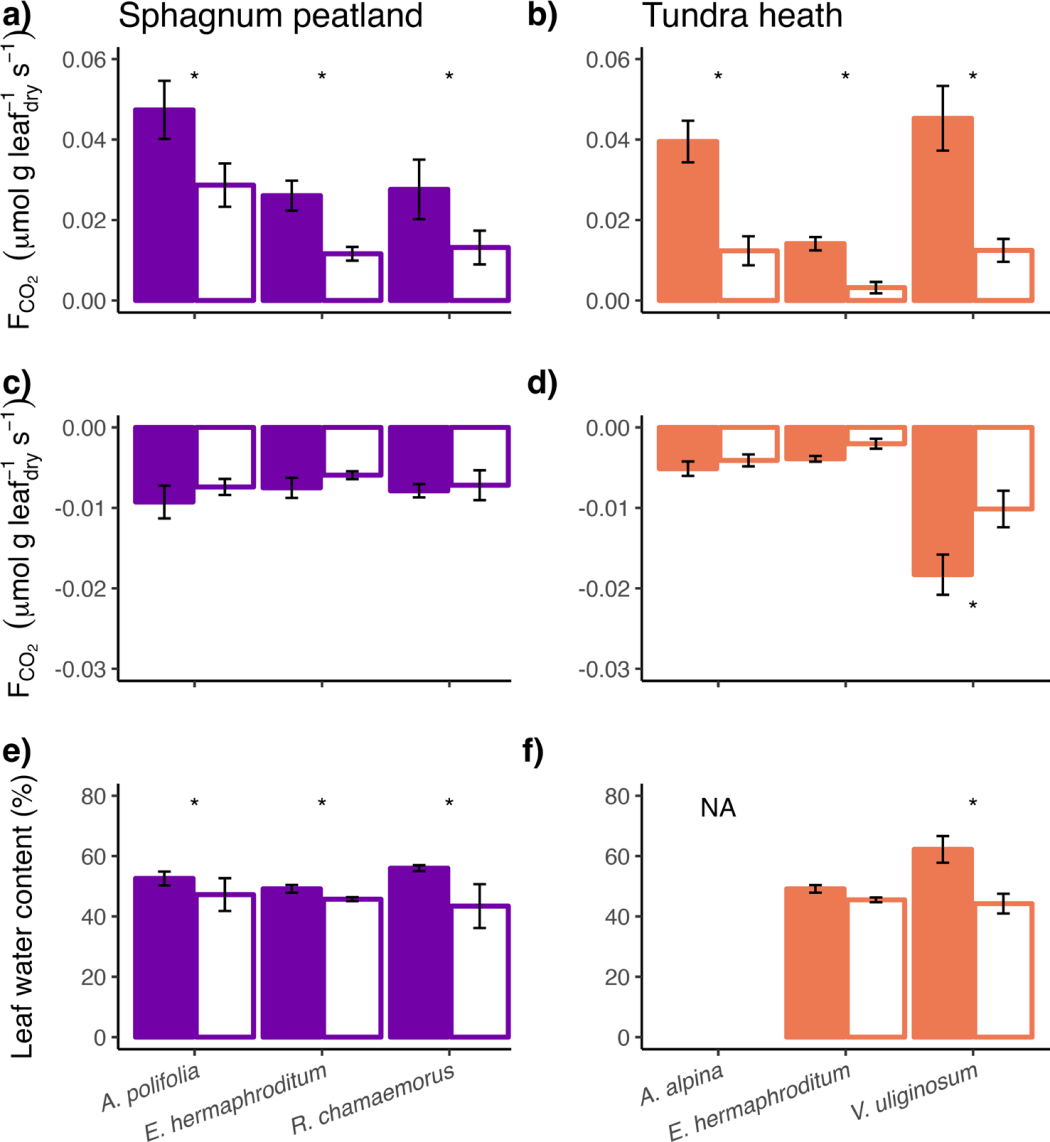
Mean leaf CO_2_ fluxes and leaf water content (± standard error, *n* = 4–8) of dominant vascular plant species in Sphagnum peatland (purple) and tundra heath (orange) under control (colored bars) and experimental drought (white bars) conditions. (a, b) Show leaf photosynthesis, (c, d) leaf respiration, (e, f) leaf water content. Measurements were taken after 35 (tundra heath) and 33 days (peatland) of experimental drought. Asterisks indicate significant differences between control and drought groups (Tukey, *p* < 0.05).

Leaf photosynthesis was on average reduced for peatland plants by 49 (±4) % and by 73 (±2) % in tundra heath. For leaf respiration, only *V. uliginosum* in tundra heath was impacted (Figure 4, Table 5). Reduction in *E. hermaphroditum* leaf fluxes was stronger in tundra heath (77%) than in peatland (55%, Figure S3). Leaf water content (Figure 4) decreased for all studied peatland species, while only *V. uliginosum* was impacted by drought in tundra heath.

**TABLE 5:** Summary of linear mixed effect model results testing the effect of treatment (control or drought) and species on leaf photosynthesis and respiration.

Response variable leaf	Ecosystem type	Drought		Species		Drought × Species	
		*F*	*p*	*F*	*p*	*F*	*p*
Photosynthesis	Peatland	12.4	0.001	7.9	0.002	0.1	0.901
	Tundra heath	40.4	< 0.001	13.6	< 0.001	3.2	0.058
Respiration	Peatland	1.8	0.188	0.9	0.436	0.1	0.869
	Tundra heath	16.4	< 0.001	45.3	< 0.001	3.8	0.036
Water content	Peatland	5.3	0.028	0.3	0.773	0.8	0.458
	Tundra heath	14.3	0.004	4.3	0.068	6.3	0.033

*Note:* For sub‐arctic *Sphagnum* peatland, the investigated species were *Andromeda polifolia* , *Empetrum hermaphroditum* , and *Rubus chamaemorus* . For tundra heath, the studied species were *Arctostaphylos alpina* , *E. hermaphroditum* , and *Vaccinium uliginosum* . Measurements were taken after 35 days (tundra heath) and 33 days (peatland) of experimental drought. numDF, denDF: photosynthesis and respiration peatland = 2, 32; photosynthesis and respiration tundra heath = 2, 26; water content peatland = 2, 32; water content tundra heath = 1, 9.

### 3.2 Plant Mortality

**FIGURE 5:**
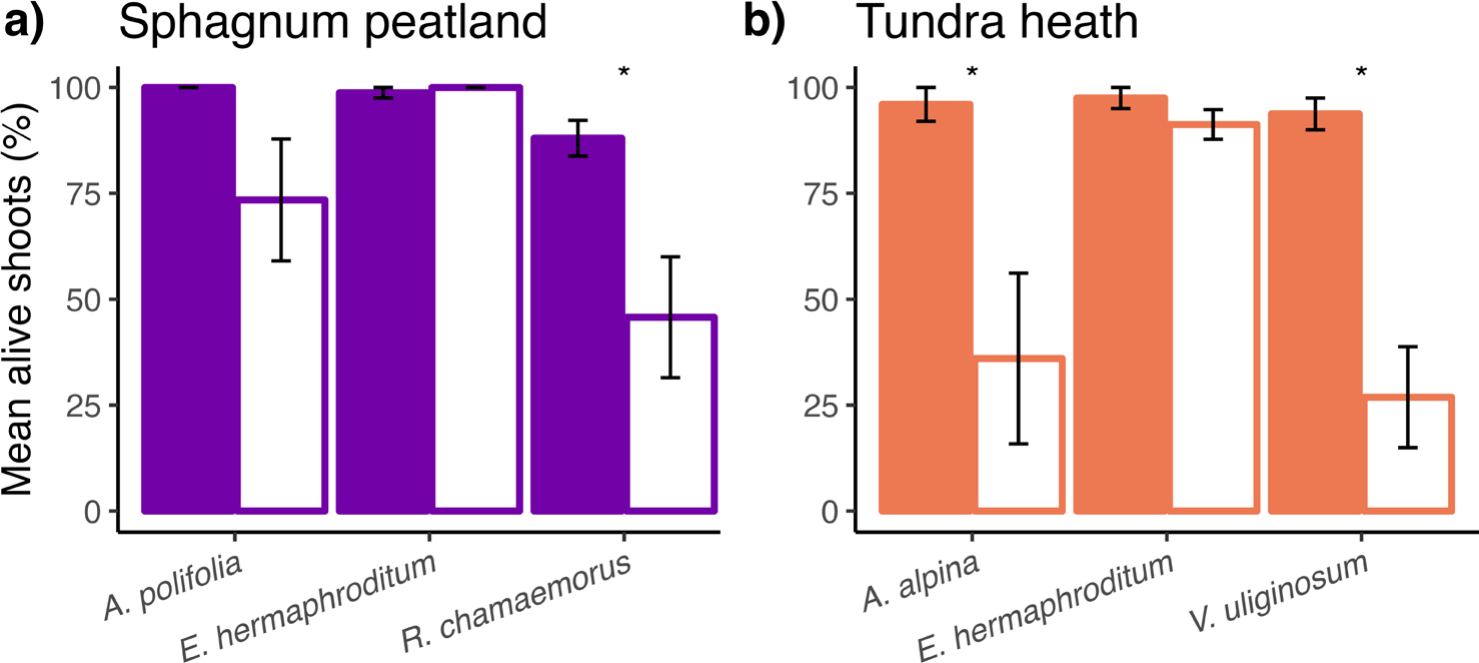
Mean alive shoots (± standard error, *n* = 8) of dominant vascular plant species after 7 weeks of experimental drought in (a) Sphagnum peatland and (b) tundra heath mesocosms under control (colored bars) and experimental drought (white bars) conditions. Asterisks indicate significant differences between control and drought groups (Tukey, *p* < 0.05).

In *Sphagnum* peatland, drought resulted in a 42% reduction in mean alive shoots for *R. chamaemorus* ; other species did not show significantly increased shoot mortality (Figure 5, Table 6). Drought impacts on mortality were more pronounced in tundra heath. The number of alive shoots of *A. alpina* and *V. uliginosum* was reduced by 60% and 67%, respectively (Table 6). *E. hermaphroditum* showed no signs of shoot mortality in either ecosystem type (Figure 5).

**TABLE 6:** Summary of linear mixed effect model results testing the effect of species and treatment on mean alive shoots in *Sphagnum* peatland and tundra heath.

Response variable	Ecosystem type	Species		Drought		Species × Drought	
		*F*	*p*	*F*	*p*	*F*	*p*
Alive shoots	Peatland	9.2	< 0.001	12.3	0.001	4.2	0.025
	Tundra heath	12.8	< 0.001	47.6	< 0.001	10.7	< 0.001

*Note:* There were 16 replicate mesocosms per ecosystem type, each with *n* = 8 allocated to control watering or to drought. Per mesocosm, 10 marked shoots per species (peatland species: *A. polifolia* , *E. hermaphroditum* , *R. chamaemorus* . Tundra heath species: *A. alpina* , *E. hermaphroditum* , *V. uliginosum* ) were visually categorized into dead or alive after 45 (peatland) and 48 (tundra heath) days of experimental drought. numDF, denDF: peatland = 2, 33; tundra heath = 2, 29.

## 4 Discussion

This study is the first to directly compare drought effects on CO_2_ fluxes between *Sphagnum* peatland and tundra heath, two key sub‐arctic ecosystem types. Simultaneous comparisons of the two ecosystem types are rare but necessary to understand carbon and water budgets at a large scale (Helbig et al. 2020; Rietze et al. 2024; Teuling et al. 2010). Furthermore, since drought events are expected to become more intense in the future (IPCC 2023), differential sensitivities, previously unaccounted for, might surface. Traditionally, research has focused on peatlands due to their large soil carbon stocks and anticipated Reco increase with warming. Our results suggest that, when considering drought, greater responses can be expected from tundra heath. Given that drought‐sensitive tundra heath covers more than half of the land area in the arctic (Ramage et al. 2024), drought events could substantially reduce summertime net CO_2_ uptake, thereby exacerbating carbon cycle feedbacks.

In agreement with our first hypothesis, drier tundra heath showed stronger reductions in CO_2_ fluxes than wetter *Sphagnum* peatland under drought (Figure 3). It is a key feature of *Sphagnum* peatlands that mosses are dominant species and soil substrate at the same time. Two of the main factors driving ecosystem drought sensitivity (plant traits and soil properties) are thus controlled by this genus. Considering that peatland mesocosms lost more water than tundra heath both in absolute and relative terms, while sustaining CO_2_ fluxes at a higher level (Figure 2), initial water content seems to play a prominent role in buffering drought responses. This is supported by the observation that drought reduced tundra heath GPP after 12 days, while for peatland GPP drought effects occurred only after 31 days (Figure 2). The former is therefore more likely to be impacted by short‐term drought events (Christian et al. 2024). Globally, surprisingly small differences in drought sensitivity have been found between biomes (Fu et al. 2022), whereas here, we can show that differential sensitivities might occur in the same landscape (Callaghan et al. 2013; Ramage et al. 2024). This highlights that studies at the scale of ecosystem types are necessary to understand drought impacts and their potential effects on large‐scale CO_2_ budgets.

In both ecosystem types, all studied vascular plant species reduced leaf CO_2_ fluxes in response to drought, supporting our second hypothesis. Since leaf CO_2_ fluxes are indicative of stomatal conductance, this supports that stomatal aperture is regulated to prevent excessive water loss and protect plants from desiccation (Hetherington and Woodward 2003; Liang et al. 2023). While the general pattern of leaf CO_2_ flux reduction and drop in leaf water content agreed between species and ecosystem types, vascular plant leaf CO_2_ fluxes in tundra heath exhibited a stronger drought response. Therefore, soil water content (Drake et al. 2017) rather than species‐specific traits (Fei et al. 2017; Teuling et al. 2010) can be seen as drivers of vascular plant response to drought. Notably, the prominent role of water content is supported by the response of *E. hermaphroditum* which occurred in both ecosystem types (Table S1) and exhibited stronger reductions in leaf fluxes and water content in tundra heath (Figures S3 and S4) than in peatland. *Sphagnum* mosses store significant amounts of water both within their cells and in extracellular spaces, particularly between their leaves (Hayward and Clymo 1982; Kokkonen et al. 2024), which likely sustains vascular plant water needs during drought and thus might increase their drought tolerance in peatlands. Thus, the combination of water retention by mosses together with the conservative water use by vascular plants in peatlands might contribute to ecosystem‐level drought resistance (cf. Anderegg et al. 2018). In contrast, drier soil conditions in tundra heath likely led to an earlier onset of plant water limitation, and leaf CO_2_ flux reductions were therefore more pronounced. These findings highlight the significant role of water retention traits typical for *Sphagnum* mosses in buffering drought impacts.

In agreement with our third hypothesis, plant mortality was higher in tundra heath than in *Sphagnum* peatland. Only deciduous plants showed mortality, which is directly relevant for ecosystem CO_2_ fluxes since they make a stronger contribution to NEE than evergreen dwarf shrubs in the arctic (Strimbeck et al. 2019; Sundqvist et al. 2020). The pronounced dieback of deciduous plants in tundra heath likely drives the greater mesocosm GPP reductions. Furthermore, mortality of dominant plant species likely represents a significant drought legacy for the subsequent growing season and could suppress GPP over a longer time. Interestingly, while no clear differences between species were observed for leaf CO_2_ fluxes, mortality was species‐specific. There was no shoot mortality for *E. hermaphroditum* in either of the ecosystems, while other species died. In contrast, *E. hermaphroditum* showed an increased mortality following extreme winter events (Bokhorst et al. 2015; Parmentier et al. 2018). With ongoing climate change, it becomes more likely that an extreme event in summer is followed by an event in winter or *vice versa*. Consequently, ecosystems might still be recovering while experiencing a follow‐up extreme event in the future. This might have legacy effects on plant communities and ecosystem CO_2_ balance. Therefore, there is an urgent need to study the effects of multiple extreme events.

It is important to note that the type of drought investigated in the current study—precipitation deficit affecting low water content—does not capture all drought scenarios. Under high evaporative demand (e.g., via high vapor pressure deficit) which is expected under future warming (Novick et al. 2016; Yuan et al. 2019), peatlands might experience greater water losses than observed in the present experiment, due to the lack of stomatal regulation in mosses. Consequently, peatlands may face stronger drought responses than currently assessed. In comparison, stomatal regulation by vascular plants in tundra heath might prevent excessive water losses via transpiration, similar to the findings by Helbig et al. (2020). Furthermore, the impacts of drought critically depend on its duration (Lund et al. 2012; Smith et al. 2024). If the experimental drought had continued, tundra heath GPP might be reduced to zero, followed by *Sphagnum* peatland as soil water content drops further. These variations highlight the need for further research into the ecosystem‐specific interactions among the drivers of drought sensitivity (atmospheric conditions, plant traits, and soil properties).

Each approach to study drought comes with certain caveats (De Boeck et al. 2015). For example, field experiments, one of the most common approaches, have been shown to underestimate drought impacts when compared to observations from natural droughts (on grass‐ and shrublands, Kröel‐Dulay et al. 2022). Conversely, field observations lack true seasonal controls, complicating attributions of drought effects (Kröel‐Dulay et al. 2022). For the comparison of ecosystem types, which was a major goal of our study, the use of mesocosms had key advantages: responses could be measured continuously and almost simultaneously under similar atmospheric conditions, which ensured the comparability of treatments and ecosystems. Additionally, soil moisture could be easily controlled together with the possibility to impose extreme drought conditions. The high survival of plants in the control group mesocosms suggests that any potential plant damage from root severance during field sampling was negligible. In agreement with that, the mesocosm flux results of the control group fall in the same order of magnitude as those reported in arctic‐wide flux summaries (Virkkala et al. 2022). However, mesocosms only represent a small part of the full soil horizon from the field (1–3 m in the Stordalen peatland, Johansson et al. 2006), and plant–soil linkages influencing CO_2_ fluxes cannot be fully assessed (e.g., Williams and de Vries 2020). We therefore emphasize relative comparisons between treatments and ecosystem types rather than focusing on absolute flux values.

Most arctic studies focus on gradual temperature increases, while our findings highlight the potential impact of drought. While warming has been shown to amplify Reco by 30% (Maes et al. 2024), drought suppressed it by 40%–53%. Given their magnitude and interactions with warming (Liang et al. 2024), drought effects may play a crucial role in shaping CO_2_ budgets. With GPP being more affected by drought than Reco, our findings are in line with drought effects on temperate ecosystems (Schwalm et al. 2010; Shi et al. 2014). This drought‐induced imbalance in CO_2_ fluxes, however, could be particularly important for the arctic, since a large part of annual GPP is concentrated within a short growing season (e.g., See et al. 2024). Furthermore, since GPP in the arctic is already water‐limited (Zona et al. 2023), it likely is sensitive to future drought events. In contrast, Reco extends throughout the entire year, with increases already observed and predicted to continue during winter (Natali et al. 2019; See et al. 2024). Due to this strong seasonality of flux components in the arctic, drought could suppress annual CO_2_ gains much more than it would be the case in temperate ecosystems with longer growing seasons (e.g., Churkina et al. 2005).

## 5 Conclusion

As drought sensitivity is ecosystem‐dependent, drought studies should include various ecosystem types to ensure reliable estimates of drought effects on CO_2_ fluxes at the biome scale. The pronounced drought effects on GPP compared to Reco might shift the carbon balance of drought‐affected areas in the arctic toward a net source, amplifying existing carbon cycle feedbacks. Our study further suggests that for an understanding of the arctic carbon‐climate feedback, not only ecosystem respiration but also reductions in productivity need to be considered. Importantly, due to the increase in mortality, drought impacts may be long‐lasting. Because of the potentially large scale to which these findings might apply, we propose to include this information in earth system models to ensure accurate carbon and water cycle predictions in the Arctic under a changing climate.

## Author Contributions

**Valentin Heinzelmann:** conceptualization, formal analysis, investigation, methodology, supervision, visualization, writing – original draft, writing – review and editing. **Julia Marinissen:** conceptualization, formal analysis, investigation, methodology. **Rien Aerts:** conceptualization, funding acquisition, methodology, supervision, writing – review and editing. **J. Hans C. Cornelissen:** conceptualization, methodology, writing – review and editing. **Stef Bokhorst:** conceptualization, funding acquisition, methodology, project administration, supervision, writing – review and editing.

## Conflicts of Interest

The authors declare no conflicts of interest.

## Supporting information


Data S1.

## Data Availability

The data that support the findings of this study are openly available from the Netherlands Polar Data Center at https://npdc.nl/dataset/ad9c6af1‐a0e5‐506f‐bd05‐27738194b89d.
