# Supplementary material for: Stronger Drought Response of CO2 Fluxes in Tundra Heath Compared to Sphagnum Peatland in the Sub‐Arctic
Source: Glob Chang Biol. 2025 Apr 24;31(4):e70210. doi: 10.1111/gcb.70210 (PMC12020989; doi:10.1111/gcb.70210)
Supplement: Supplementary file 1 — Data S1. [file GCB-31-e70210-s001.docx]

Supporting information for

**Stronger drought response of CO_2_ fluxes in tundra heath compared to *Sphagnum* peatland in the sub-Arctic**

Authors: Valentin Heinzelmann*, Julia Marinissen, Rien Aerts, J. Hans C. Cornelissen, Stef Bokhorst

Vrije Universiteit Amsterdam, Amsterdam Institute for Life and Environment, Section Systems Ecology
Amsterdam, The Netherlands

*Corresponding author: Valentin Heinzelmann, email: v.p.heinzelmann@vu.nl


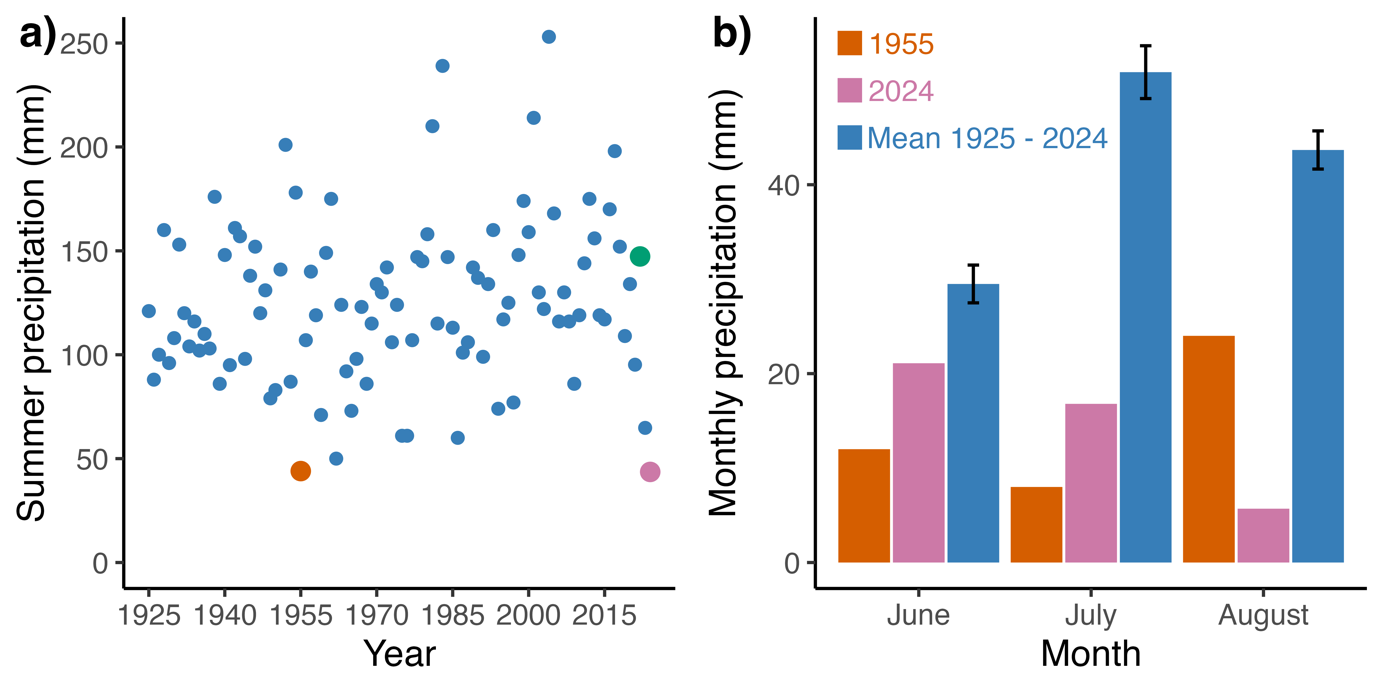


Figure S 1: a) Summer precipitation sums (June, July, August) in Abisko (1925 - 2024). Drought summers in 1955 and 2024 are marked in orange and magenta, respectively. The year 2022 is marked in green. b) Monthly precipitation in drought years 1955, 2024, and mean precipitation (± standard error, n = 100) between 1925 - 2024. Dataset is combined from ANS (2021) and SMHI (2025).


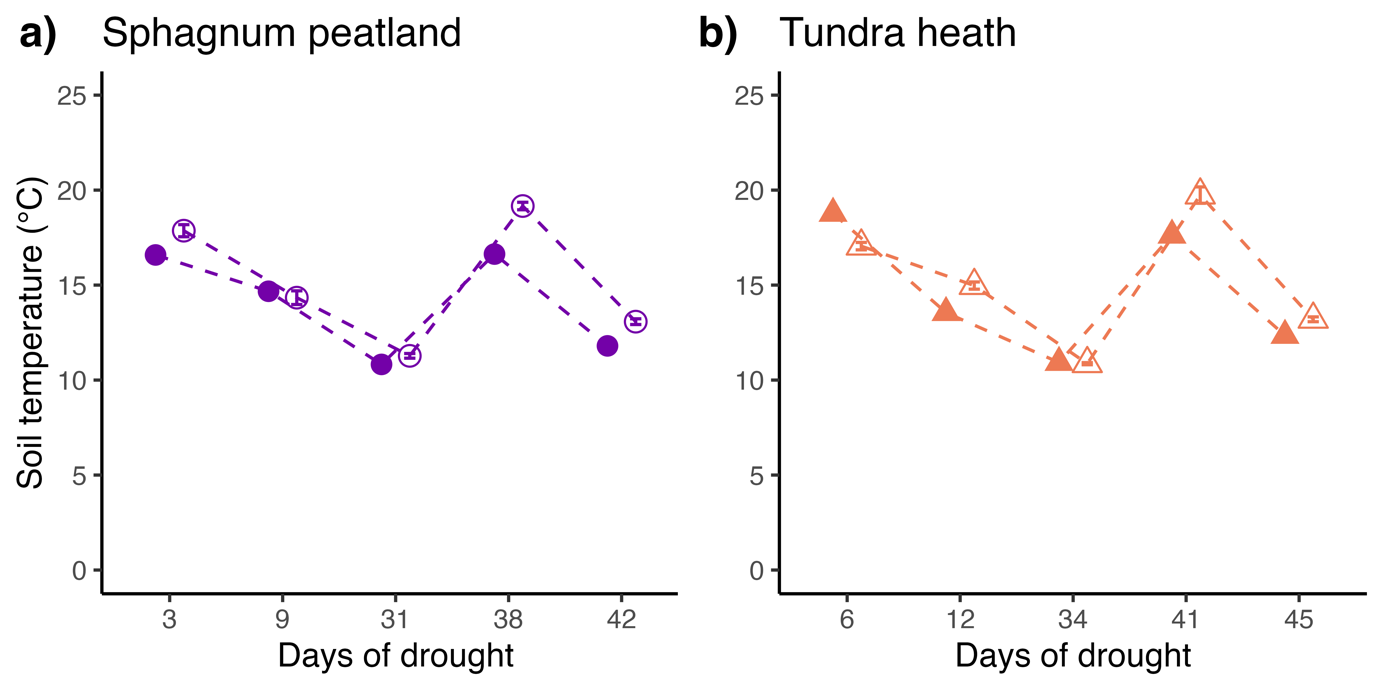


Figure S 2: Mean soil temperature (± standard error, n = 8) under control (colored circles or triangles) and experimental drought (white circles or triangles) conditions in a) Sphagnum peatland and b) tundra heath mesocosms.


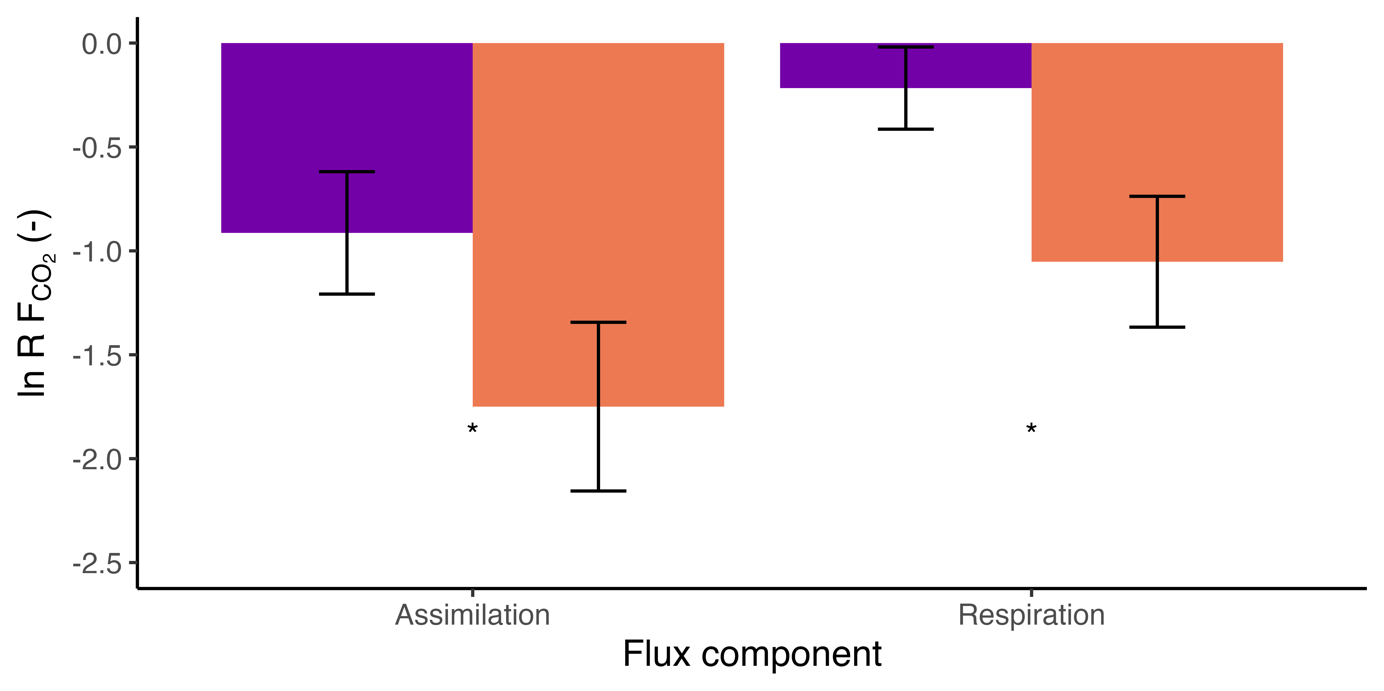


Figure S 3: Mean Ln response ratio (± standard error, n = 7 to 8) of E. hermaphroditum leaf fluxes. Shown are flux components on Sphagnum peatland (purple) and on tundra heath (orange). Measurements were taken after 35 (tundra heath) and 33 days (peatland) of experimental drought. Ecosystem type F_1,19_ = 7.4, p = 0.014, flux component F_1,19_ = 5.7, p = 0.028, Ecosystem type x flux component F_1,19_ = 0, p = 0.999. Asterisks indicate significant differences between control and drought groups (Tukey, p < 0.05).


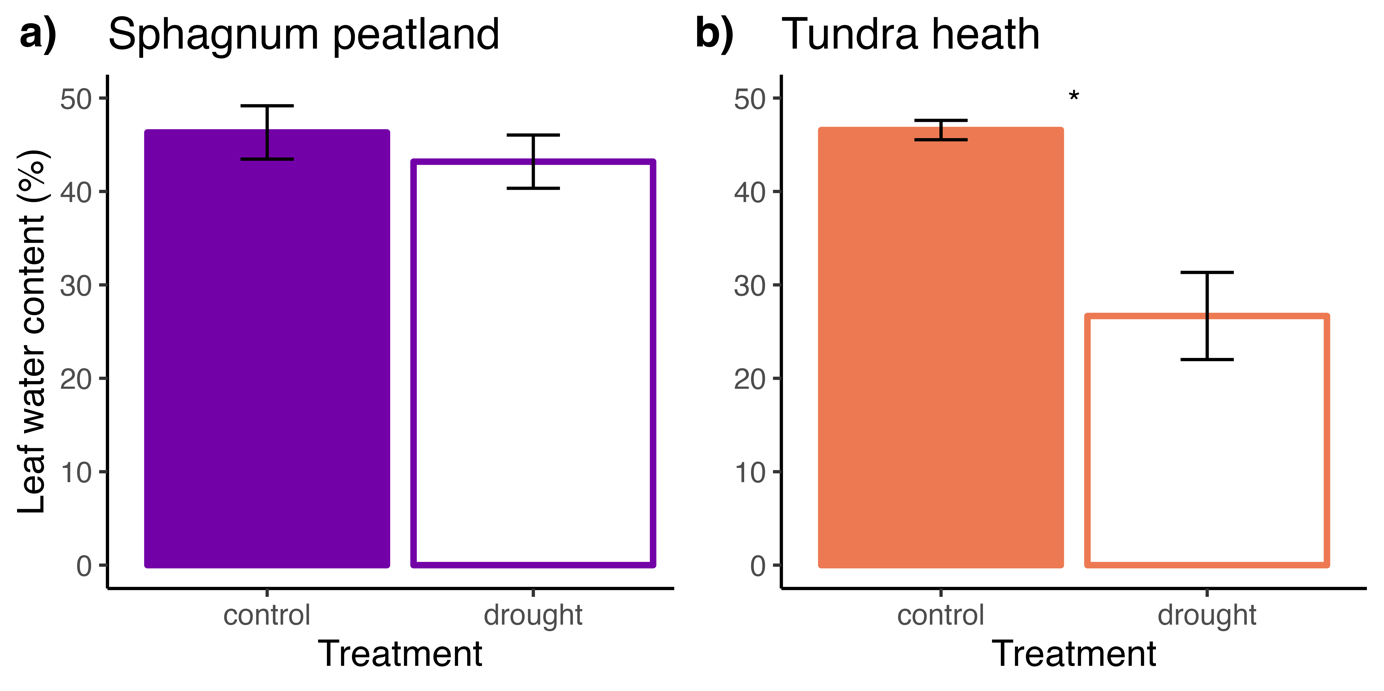


Figure S 4: Mean leaf water content (± standard error, n = 8) of E. hermaphroditum after 45 (peatland) and 48 (tundra heath) days of experimental drought in a) Sphagnum peatland and b) tundra heath mesocosms under control (colored bars) and experimental drought (white bars) conditions. Treatment F_1,20_ = 13.4, p = 0.002, ecosystem type F_1,20_ = 7.1, p = 0.015, treatment x ecosystem type F_1,20_ = 7.1, p = 0.015. Asterisk indicates significant difference between control and drought group (Tukey, p < 0.05).

Table S 1: Mean point-intercept hits per vascular plant species (± standard error, on n = 16 mesocosms per ecosystem type Sphagnum peatland and tundra heath). The point intercept method (Jonasson, 1988) was used in the first week of August 2022. Species abundance has been recorded at a total of 28 locations per mesocosm (with a surface area of 39 cm x 28 cm).

| *Sphagnum* peatland | | Tundra heath | |
| --- | --- | --- | --- |
| **Species** | **Mean hits** | **Species** | **Mean hits** |
| *E. hermaphroditum* | 14.56 (±1.84) | *E. hermaphroditum* | 28.94 (± 2.60) |
| *R. chamaemorus* | 7.75 (± 0.85) | *E. arvense* | 6.94 (± 1.27) |
| *bare ground* | 5.81 (± 0.65) | *A. alpina* | 4.50 (± 1.25) |
| *V. microcarpum* | 4.88 (± 0.90) | *A. polifolia* | 4.44 (± 0.68) |
| *A. polifolia* | 2.81 (± 0.60) | *V. uliginosum* | 4.31 (± 0.77) |
| *graminoid* | 2.38 (± 0.91) | *graminoid* | 3.06 (± 0.92) |
| *V. uliginosum* | 0.88 (± 0.41) | *bare ground* | 1.94 (± 0.43) |
| *B. nana* | 0.50 (± 0.33) | *V. vitis-idaea* | 1.50 (± 0.66) |
| *V. myrtillus* | 0.31 (± 0.18) | *R. lapponicum* | 1.44 (± 0.66) |
| *P. vulgaris* | 0.12 (± 0.12) | *Salix spec.* | 0.31 (± 0.31) |
| *V. vitis-idaea* | 0.06 (± 0.06) | *B. nana* | 0.25 (± 0.14) |
|  |  | *M. orchis* | 0.12 (± 0.09) |

Table S 2: Summary of linear mixed effect model results testing the effect of ecosystem type (Sphagnum peatland or tundra heath), flux component (GPP or Reco) and Ln response ratio of water content on the Ln response ratio of mesocosm CO_2_ fluxes. There were 16 replicate mesocosms per ecosystem type, each with n = 8 allocated to control watering or to drought. Fluxes were measured 5 times during the experiment.

| **Response variable** | | **Variable** | **F** | **p** |
| --- | --- | --- | --- | --- |
| Ln response ratio CO_2_ fluxes | | Ecosystem type | 13.4 | < 0.001 |
|  | | Flux component | 12.7 | < 0.001 |
|  | | Ln response water content | 124.1 | < 0.001 |
|  | | Ecosystem type x Component | 2.8 | 0.095 |
|  | | Ecosystem type x Ln response water content | 13.3 | < 0.001 |
|  | | Flux component x Ln response water content | 11.3 | 0.001 |
|  | | Ecosystem type x Flux component x Ln response water content | 3.6 | 0.061 |
| numDF, denDF: = 1, 143 |  |  |  |  |
